# Therapeutic Application of Phage Capsule Depolymerases against K1, K5, and K30 Capsulated *E. coli* in Mice

**DOI:** 10.3389/fmicb.2017.02257

**Published:** 2017-11-16

**Authors:** Han Lin, Matthew L. Paff, Ian J. Molineux, James J. Bull

**Affiliations:** ^1^Department of Integrative Biology, The University of Texas at Austin, Austin, TX, United States; ^2^Institute for Cellular and Molecular Biology, The University of Texas at Austin, Austin, TX, United States; ^3^Department of Molecular Biosciences, The University of Texas at Austin, Austin, TX, United States; ^4^Center for Computational Biology and Bioinformatics, The University of Texas at Austin, Austin, TX, United States

**Keywords:** bacterial capsule, phage, capsule depolymerase, infection, antibiotic

## Abstract

Capsule depolymerase enzymes offer a promising class of new antibiotics. *In vivo* studies are encouraging but it is unclear how well this type of phage product will generalize in therapeutics, or whether different depolymerases against the same capsule function similarly. Here, *in vivo* efficacy was tested using cloned bacteriophage depolymerases against *Escherichia coli* strains with three different capsule types: K1, K5, and K30. When treating infections with the cognate capsule type in a mouse thigh model, the previously studied K1E depolymerase rescued poorly, whereas K1F, K1H, K5, and K30 depolymerases rescued well. K30 gp41 was identified as the catalytically active protein. In contrast to the *in vivo* studies, K1E enzyme actively degraded K1 capsule polysaccharide *in vitro* and sensitized K1 bacteria to serum killing. The only *in vitro* correlate of poor K1E performance *in vivo* was that the purified enzyme did not form the expected trimer. K1E appeared as an 18-mer which might limit its *in vivo* distribution. Overall, depolymerases were easily identified, cloned from phage genomes, and as purified proteins they proved generally effective.

## Introduction

Both intact phages and their proteins are promising therapies for antibiotic resistant bacteria (Lewis, 2013; Drulis-Kawa et al., 2015; Abedon et al., 2017) especially given the current slow pace of new antibiotic discovery (Silver, 2011). Phage therapy’s advantages include high host specificity, amplification where bacteria are dense, an abundance and diversity of wild phages, and evolution in response to bacterial resistance (Weber-Dabrowska et al., 2016). Yet there are also drawbacks, such as the need to match phages to the infecting strain and the simple fact that bacteria have many mechanisms of escape.

The use of intact phages to treat infections is an old concept; the first phage therapy in humans was attempted in 1919 (D’Herelle and Smith, 1926; Sulakvelidze et al., 2001) and the first report of clinical phage therapy was published in 1921 (Bruynoghe and Maisin, 1921). More recently, it has been realized that therapy may utilize phage proteins instead of intact phages. These alternative technologies have many advantages of phages – an abundant and diverse collection of phage proteins occur in nature, evolved specifically to act against bacteria, and they potentially overcome one of the main drawbacks of traditional phage therapy, namely the narrow specificity of phages. Thus, Gram-positive phage endolysins also lyse Gram-positive bacteria from the outside and have far broader host ranges than do individual phages (Fischetti, 2011; Pastagia et al., 2013; Nakonieczna et al., 2015; Pires et al., 2016). Mycobacterial phage endolysins have activity against mycobacteria when added to cells (Payne and Hatfull, 2012; Grover et al., 2014), and combining an endolysin with a cell-permeating peptide (Artilysin^®^) also shows promise for disrupting the complex cell envelope of Gram-negative bacteria (Briers et al., 2014; Gerstmans et al., 2016; Pires et al., 2016). Phage-encoded polysaccharide depolymerases, which potentially also have a broad host range, can degrade carbohydrate barriers on bacterial cell surfaces such as capsule, lipopolysaccharide and biofilm matrix to compromise bacterial virulence (Latka et al., 2017). Capsule depolymerases are one class of polysaccharide depolymerases that can strip capsules and thereby expose bacteria to immune attack, with the further advantage that the bacteria are not lysed and thus do not release endotoxins (Azeredo and Sutherland, 2008).

A bacterial capsule is a thick polysaccharide layer found on many bacteria. Over 80 different types of *Escherichia coli* capsules have been identified and classified into four groups, based on their varied serological, biochemical and genetic properties (Orskov et al., 1977; Whitfield, 2006). Our work involves K1 and K5 capsules in Group 2, two types highly frequently found in extra-intestinal infection (Orskov et al., 1977), and the K30 capsule in Group 1, a type found in intestinal infection and well-studied for capsule biosynthesis (Whitfield, 2006). Characterization of capsules surrounding other bacteria has revealed both the same and novel structures but nomenclature is often specific for a particular genus (Orskov et al., 1977; Roberts, 1996). Possible functions of capsules include protecting bacteria from desiccation, bacterial adherence to surfaces and to each other, helping bacteria escape complement-mediated killing or phagocytosis, and resisting immune response (Roberts, 1996).

Phages that grow on capsulated strains commonly encode tailspike enzymes that degrade the capsule, providing a ready source of enzymes. Some capsule depolymerases assemble as trimers with the help of a C-terminal domain that functions as a chaperone, which is then autoproteolytically cleaved (Gerardy-Schahn et al., 1995; Muhlenhoff et al., 2003; Schwarzer et al., 2007, 2012; Leiman and Molineux, 2008). One of the depolymerases used in this work, K1E, assembles on the phage virion using an adaptor protein, which in addition likely contributes to accurate trimerization (Tomlinson and Taylor, 1985; Gerardy-Schahn et al., 1995; Stummeyer et al., 2006). However, other tailspike enzymes, e.g., P22 gp9, autonomously fold as a trimer and spontaneously assemble correctly on a mature phage head, although a cellular chaperone may improve efficiency (Brunschier et al., 1993).

This study tests capsule depolymerases as therapeutic agents against capsulated bacteria. The few experimental studies of phage depolymerase treatments in rodents, including those with the K1E depolymerase in a neonatal rat infection model, have met with apparent success (Mushtaq et al., 2004, 2005; Lin et al., 2014; Pan et al., 2015), but the generality and wide-scale technical feasibility of the approach remains unclear because few types of capsules and depolymerases have been tested. Further, different infection models have been used, and different enzymes degrading the same capsule have not been compared side-by-side. Further evidence of depolymerase efficacy is offered here for five different phage depolymerases against three capsule types in a mouse infection model.

## Materials and Methods

### Strains and Culture Conditions

The *E. coli* strains RS218 (O18:K1:H7) (Achtman et al., 1983), ATCC 23506 (O10:K5(L):H4), and E69 (O9:K30) (Orskov et al., 1977) were used for mouse infection, capsule isolation and serum sensitivity assays. *E. coli* BL21(DE3) was used for protein expression and purification. Bacteria were grown in LB (10 g tryptone, 5 g yeast extract, 10 g NaCl per liter) broth in 37°C shakers unless otherwise noted. The concentration of viable bacteria was determined by colony counts using LB agar (1.3% w/v) plates.

The *E. coli* strain EV36 (Vimr and Troy, 1985) was used to propagate the coliphages K1E, K1F, and K1H (Bull et al., 2010). *E. coli* ATCC 23506 and E69 were used for propagation, respectively, of the coliphages K1-5 (Scholl et al., 2001, 2004) and K30 (Whitfield and Lam, 1986). Phages were grown and purified as previously described (Scholl et al., 2001; Leiman et al., 2007). Briefly, the coliphages were added to bacterial cultures at an OD_600_ = 0.25–0.4 at a multiplicity of infection of 4, followed by incubation at 37°C with aeration until the culture cleared. Phages were precipitated with 0.5 M NaCl and 10% PEG 8000, and then purified by equilibrium CsCl gradient centrifugation in SM buffer (50 mM Tris–HCl, 100 mM NaCl, 8 mM MgSO_4_, pH 7.5) supplemented with CsCl to a density of 1.5 g/ml. After dialysis into SM buffer using 12–14 kDa MWCO dialysis membranes (Spectrum), phage titers were determined by plaque counts using a LB soft agar (0.65%) overlay.

### Cloning of Phage Capsule Depolymerase Genes

Phage genomic DNA was extracted as described for phage λ (Sambrook et al., 1989), and then was used as template to amplify the depolymerase genes K1E, K1F, K1H, K5 or those for the putative enzymes K30 gp41 and K30 gp42. Gene information and the PCR primers are listed in Supplementary Table S1. PCR products were then assembled into NdeI- and EcoRI-digested pET28b (EMD Biosciences Inc.) using the Gibson Assembly Master Mix (NEB Inc.).

### Protein Expression and Purification

After overexpression of their genes, capsule depolymerases were purified essentially as previously described (Leggate et al., 2002). Briefly, the pET28b derivatives were transformed into *E. coli* BL21(DE3). Expression of the recombinant His-tagged depolymerase genes was induced at *A*_600_ = 0.6 with 0.5 mM isopropyl-β-D-thiogalactopyranoside (IPTG) followed by overnight growth at 20°C. Cells were lysed by sonication in lysis buffer (50 mM Na_2_HPO_4_, 300 mM NaCl, 10 mM imidazole, pH 7.5) and the depolymerases were purified using HisPur Ni-NTA resin (Thermo Fisher Scientific Inc.) according to the user guide. After dialysis into phosphate-buffered saline (PBS) buffer (137 mM NaCl, 2.7 mM KCl, 10 mM Na_2_HPO_4_, 1.8 mM KH_2_PO_4_, pH 7.5) using 3.5 kDa MWCO dialysis membranes (Spectrum), the depolymerases were used directly in all experiments. Protein concentrations were estimated by *A*_280_, using a Nanodrop ND-1000.

Purified proteins were analyzed by SDS-PAGE and size exclusion chromatography. SDS-PAGE was performed using a 10% resolving gel with a 4% stacking gel. Proteins were denatured at 100°C for 5 min. After electrophoresis, proteins were stained with Coomassie brilliant blue. Size exclusion chromatography was performed on an AKTA FPLC (GE Healthcare) at 4°C. Depolymerases, in 25 mM sodium phosphate, 150 mM NaCl, pH 7.5, were applied to a Superose 6 10/300 GL column (GE Healthcare). Elution was at a flow rate of 0.4 ml/min and proteins were detected at *A*_280_. Molecular weights were estimated using the high molecular weight gel filtration calibration kit (GE Healthcare).

### Mouse Infections and Depolymerase Treatment

Mouse work conformed to NIH guidelines and the University of Texas IACUC protocol approval (AUP-2015-00035). 4–6 weeks old female NIH Swiss outbred mice (Envigo Inc.) weighing 20–25 g were used. For infections, 1 – 4 × 10^8^ CFU (colony forming units) of bacteria in up to 100 μl were injected into the left thigh (Smith and Huggins, 1982; Bull et al., 2012; Ponnusamy et al., 2016). Doses ranged from 1.2 to 3.5 × 10^8^ CFU for *E. coli* RS218, 1.7 – 3.7 × 10^8^ CFU for *E. coli* ATCC 23506, and 1.0–3.7 × 10^8^ CFU for *E. coli* E69. The lower end of the dose range may be near a threshold that enables viability, e.g., infection by 1.2 × 10^8^ CFU of *E. coli* ATCC 23506 allowed 2 of 3 control mice to survive, whereas doses above 1.7 × 10^8^ CFU were routinely inimical to survival.

Treatment was performed by injecting a depolymerase appropriate for the capsule type into the right thigh within 0.5 h after the bacterial injection, i.e., K1E, K1F, or K1H depolymerase with *E. coli* RS218, K5 depolymerase with *E. coli* ATCC 23506, K30 gp41 or K30 gp42 with *E. coli* E69. Different doses were obtained by dilution of the stock depolymerase into PBS to yield 100 μl for an injection. The effective doses were first determined by giving 3 mice each dose (0, 2, 5, or 20 μg). Optimal doses were then used with more mice to allow statistical analysis of therapeutic efficacy. Mice were monitored at least twice daily for 5 days, and moribund mice were euthanized. The numbers of surviving mice at Day 5 were plotted, and Fisher’s Exact Test was used to evaluate the therapeutic efficacy of a depolymerase. Using SPSS software, Kaplan–Meier survival curves (Rich et al., 2010) were plotted to show the cumulative probability of survival over the 5-day period, using the Log Rank test or generalized Wilcoxon test for statistics.

To assess potential acute toxicity from the depolymerase, 3–5 mice were injected with 100 μg of depolymerase (in 100 μl PBS) or 100 μl PBS alone in the right thigh, in the absence of bacterial infection. Mice were monitored for 5 days for survival; behavior and daily body weights were measured. Statistics of the body weight gains over 5 days were performed by mixed ANOVA with repeated measures using SPSS software.

### Capsule Polysaccharide Isolation and Assay

*Escherichia coli* RS218, ATCC 23506 and E69 were grown overnight at 37°C in defined medium (10 g Casamino Acids, 10 g glucose, 12.5 g Na_2_HPO_4_⋅2H_2_0, 0.9 g KCl, and 0.6 g MgSO_4_⋅7H_2_0 per liter). Isolation of K1, K5, or K30 type capsule used extraction with pyridine acetate as previously described (Pelkonen et al., 1988). Capsules were dissolved in sterile water and stored at 4°C. The capsule concentrations were quantified by the phenol-sulfuric acid method (Dubois et al., 1956), using glucose to generate standard curves.

Degradation of capsules was monitored by gel electrophoresis followed by alcian blue staining (Møller et al., 1993; Pan et al., 2013). 10–20 μg of capsule was mixed with serial dilutions of corresponding depolymerase and incubated at 37°C for 1 h (K1E, K1F, or K1H depolymerase with K1 capsule; K5 depolymerase with K5 capsule; K30 gp41 or K30 gp42 with K30 capsule). Reactions were loaded on 12% TBE-PAGE (Tris-Boric acid-EDTA polyacrylamide gel electrophoresis) essentially as previously described (Pelkonen et al., 1988). XC (xylene cyanol), BPB (bromophenol blue) and PR (phenol red) were used together with all blue protein standards (Bio-Rad Inc.) as tracking dyes and molecular weight markers.

Quantitative analyses of depolymerase activity were performed by incubating 30–45 μg of capsule with serial dilutions of depolymerase, or by incubating 10 μg/ml depolymerase with serial dilutions of capsule, both for 30 min at 37°C, followed by determination of reducing sugar using the modified dinitrosalicylic acid (DNSA) reagent (Miller, 1959). Glucose served as the standard. The quantity of reducing sugar released against enzyme concentration was plotted, and enzyme specific activity is expressed as nmol glucose equivalents released per min per mg protein (McCallum et al., 1989). Hanes–Woolf plots (*a*/*v* against *a*, where a is the capsule concentration and *v* is the reaction velocity) allowed the determination of kinetic parameters, assuming K1 capsule at a molecular weight of 54 kDa (Hallenbeck et al., 1987; Leggate et al., 2002).

### Serum Sensitivity Assay

The assay was adapted from previous work (Podschun et al., 1993; Fang et al., 2004; Mushtaq et al., 2004). Briefly, 4–6 × 10^7^ CFU/ml of log phase bacteria were incubated with or without depolymerase (100 μg/ml) for 1.5 (K1, K5) or 2 h (K30) at 37°C. Mixtures were diluted and 4–6 × 10^4^ cells were incubated with 75% of human serum (Sigma–Aldrich Inc.), heat inactivated serum (56°C, 30 min) or PBS for 1.5 (K5) or 2 h (K1, K30) at 37°C and then plated to determine CFU. Assays were repeated at least three times, and Student’s *t*-test with an appropriately adjusted degree of freedom was used to evaluate the enzyme’s effect in sensitizing cells to serum killing by the survival ratio X(serum)/X(PBS), where *X* is CFU/ml. The null hypothesis is that one treatment is the same as the other, or X1(serum)/X1(PBS) = X2(serum)/X2(PBS), i.e., log [X1(serum)] – log[X1(PBS)] – {log [X2(serum)] – log[X2(PBS)]} = 0.

## Results

### Expression and Purification of Recombinant Depolymerases

Depolymerases from phages K1E, K1F, K1H, and K5 were purified from expression plasmids of previously identified genes (Petter and Vimr, 1993; Long et al., 1995; Clarke et al., 2000; Machida et al., 2000; Muhlenhoff et al., 2003; Scholl et al., 2004). K30 depolymerase was described as a trimer of a heterodimer of 90 and 52 kDa proteins (McCallum et al., 1989). Inspection of the subsequently deposited and annotated K30 genome sequence (Genbank NC_015719), which is largely syntenic with the well-characterized T7 genome, revealed only two likely candidate genes for the proteins: the K30 gene *42* product is a putative lipase/acylhydrolase, and gene *41* is a putative tailspike. Expected sizes of both proteins correspond to subunits of the depolymerase originally characterized biochemically. Both genes were therefore cloned into expression plasmids and all the His-tagged proteins were purified, yielding approximately 10 mg of K5 or K30 gp41 per liter culture, 20 – 30 mg per liter of K1E, K1F, or K1H, and 40 mg per liter of K30 gp42. We therefore expressed and tested a total of six proteins, including four depolymerases and two putative depolymerases.

**FIGURE 1:**
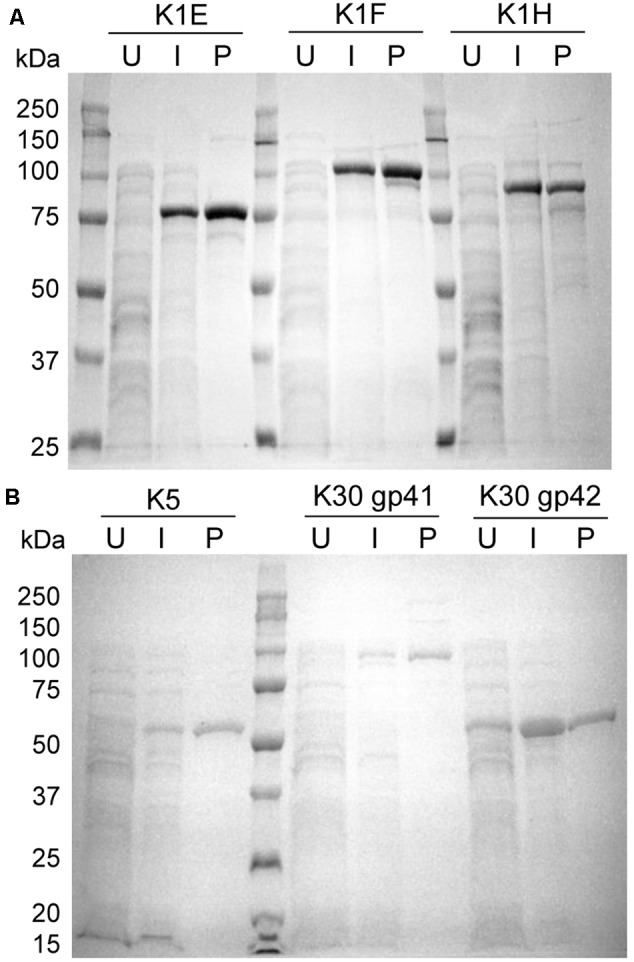
Expression and purification of recombinant K1, K5, and K30 depolymerases. **(A)** SDS-PAGE showing induced expression and purification of K1E, K1F, and K1H depolymerases. 50 μg of uninduced or induced whole cell lysate obtained after sonication, or 2 μg of enzyme purified was fractionated by SDS-PAGE followed by Coomassie Blue staining. U, uninduced whole cell lysate; I, induced whole cell lysate; P, purified enzyme. **(B)** SDS-PAGE of K5 and putative K30 depolymerases after a similar induction and protein purification.

The affinity-purified K1 and K5 depolymerases migrated on SDS-PAGE in accordance with their expected sizes after proteolysis (K1E 76 kDa, K1F 103 kDa, K1H 93 kDa and K5 52 kDa) (**Figure 1**). Sizes of the two K30 proteins were estimated to be 97 and 57 kDa, suggesting that they are not post-translationally cleaved. By densitometry analysis all purified proteins appeared >90% pure in SDS-PAGE.

### Capsule Depolymerases Can Be Effective Therapeutics

**FIGURE 2:**
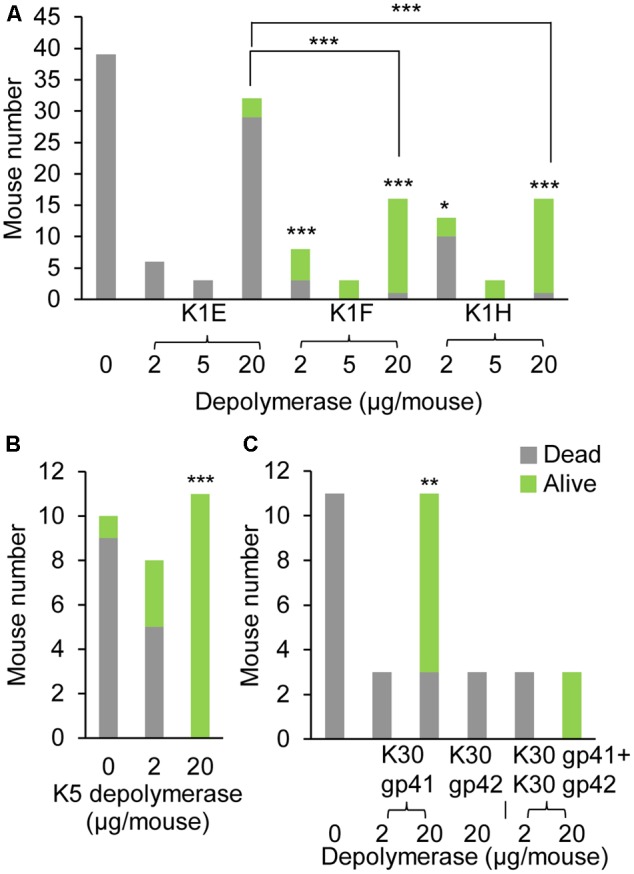
Mouse survival at Day 5 post infection and depolymerase treatment. 1.2–3.5 × 10^8^ CFU of *Escherichia coli* RS218 **(A)**, 1.7–3.7 × 10^8^ of *E. coli* ATCC 23506 **(B)**, or 1.0–3.7 × 10^8^ of *E. coli* E69 **(C)** were injected to the left thigh, followed by corresponding depolymerase injection in the right thigh at various doses. Three mice each dose were preliminarily tested for dose titration, then more mice were repeated at select doses to validate the treatment efficacy. Mouse survival was monitored for 5 days. The numbers of surviving and dead mice at day 5 were evaluated by Fisher’s Exact Test for treatments with *n* >= 8: ^∗^*p* < 0.05, ^∗∗^*p* < 0.01, ^∗∗∗^*p* < 0.001 compared to control or as noted.

**FIGURE 3:**
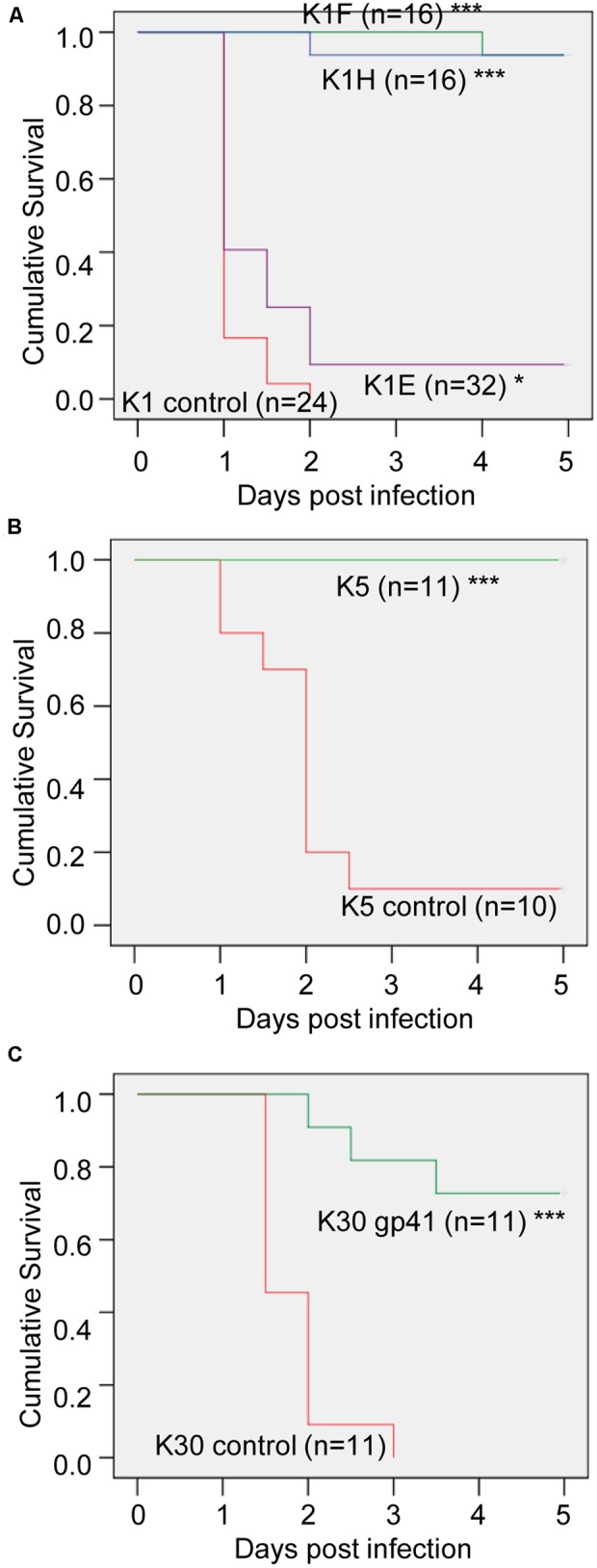
Mouse survival curves of depolymerase treatment. Kaplan–Meier survival curves of the 20 μg-K1 **(A)**, K5 **(B)**, or K30 **(C)** depolymerase treated mice and control mice in **Figure 2** were plotted with the cumulative probability of survival over 5 days. The total mouse number n of each treatment is labeled by each curve. Log Rank test or generalized Wilcoxon test: ^∗^*p* < 0.05, ^∗∗∗^*p* < 0.001 compared to control.

Capsule depolymerases were tested in a mouse thigh model of infection. Without treatment, infection was usually lethal, whereas most mice were rescued by treatment when the enzyme dose was 20 μg per mouse, i.e., 0.8–1 mg/kg weight (**Figures 2, 3**). The exception was the K1E enzyme, which rescued only 3 of 32 mice at a dose of 20 μg per mouse (**Figures 2A, 3A**). Preliminary trials of the three K1 enzymes at lower doses suggested the effective doses of K1F and K1H were between 2 μg (both partially rescuing) and 5 μg (both rescuing 3 of 3 mice) per mouse (**Figure 2A**).

For K5, the effective dose was between 2 and 20 μg per mouse (**Figures 2B, 3B**). Of the two putative K30 depolymerases, only K30 gp41 rescued mice and then only at the higher dose tested (20 μg per mouse) (**Figures 2C, 3C**). A mixture of both K30 gp41 and K30 gp42 yielded the same survival outcome as K30 gp41 alone (**Figure 2C**), although the small sample size limits a statistical resolution. K30 gp41 appears somewhat less effective than K5 and two of the K1 enzymes.

To evaluate potential acute toxicity from enzyme injection, mice received 100 μg of depolymerase in the right thigh and were monitored for survival, behavior and body weight gains for 5 days. All the mice survived and appeared healthy without any behavior change observed. Statistics by ANOVA indicates no significant difference in body weight gains of the treated mice compared to that of the control mice receiving PBS injection (Supplementary Figure S1). These indicate no or little toxicity from enzyme injection.

### *In Vitro* Depolymerase Assays

The depolymerases were generally effective for the *in vivo* treatment, but different enzymes also showed differences in efficacy, especially K1E, which was a relatively poor therapeutic agent. K30 gp42 had no effect and likely lacks depolymerase activity. Therefore, *in vitro* assays were conducted to directly assess enzyme activities.

**FIGURE 4:**
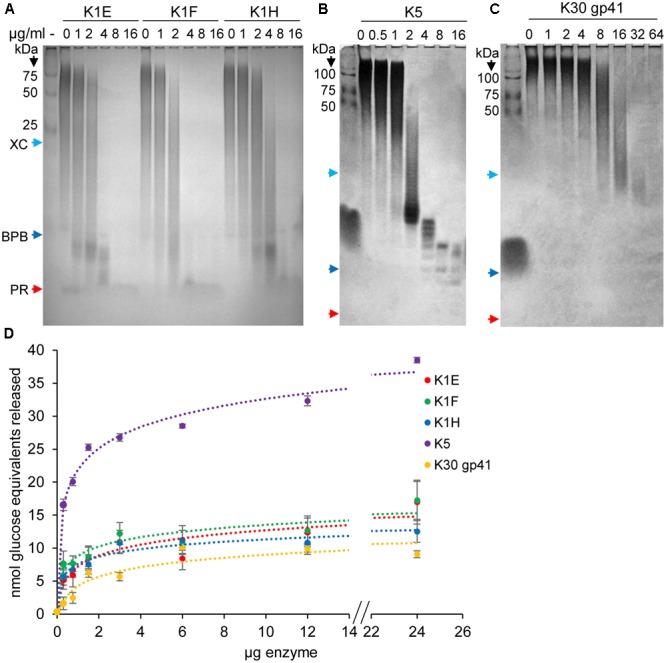
Capsule degradation by depolymerases. **(A)** 10–20 μg of K1 capsule was incubated with serial dilutions of K1E, K1F, or K1H depolymerase at 37°C for 1 h, and then fractionated using 12% TBE-PAGE gel followed by Alcian Blue staining. Protein standards and dyes (XC, xylene cyanol FF; BPB, bromophenol blue; PR, phenol red) were loaded as molecular weight markers. Similar assays were performed using K5 **(B)** or putative K30 **(C)** depolymerase and their respective capsules. **(D)** Quantitative assay of capsule degradation by depolymerases. 30–45 μg of capsule was incubated with ranged doses of depolymerase for 30 min at 37°C. The product of reducing sugar was quantified by dinitrosalicylic acid (DNSA), with glucose as standard. The calculated amount of reducing sugar (nmol glucose equivalents) was plotted against enzyme doses.

Activities of the different K1 enzymes were compared using a gel assay to monitor capsule degradation. Apparent complete degradation (by visual inspection) of 10–20 μg of K1 capsule in 1 h was achieved by 4–8 μg/ml of K1E or K1F depolymerase, and by 8–16 μg/ml of the K1H enzyme (**Figure 4A**). Two μg/ml of K1E or K1F enzyme completely degraded the capsule within 3 h, but K1H was again significantly less effective (data not shown). Thus, both K1E and K1F perform better than K1H during *in vitro* capsule degradation, in contrast to *in vivo* therapeutic efficiencies.

K1E depolymerase activity was quantified by assaying reducing sugar release from capsule (**Figure 4D** and Supplementary Table S2). Assays with 30 μg of K1 capsule and varying K1 enzyme doses showed similar kinetics of the three K1 enzymes, with similar specific activities at lower enzyme doses (K1E 80 – 240, K1F 80 – 360, K1H 70 – 260 nmol glucose equivalents released per min per mg protein) (**Figure 4D**). Assays with 10 μg/ml of depolymerase and varying K1 capsule concentrations showed slightly better binding affinity for K1E [*K*_M_ = 4.16 μM, similar to previous reports (Long et al., 1995; Leggate et al., 2002)] than K1F and K1H, and higher catalytic efficiency than K1H (Supplementary Table S2).

Similar assays were performed with K5 and K30. 10–20 μg of K5 capsule was degraded in 1 h by 4–8 μg/ml K5 depolymerase (**Figure 4B**). Of the two putative K30 depolymerases, 64 μg/ml K30 gp41 was required to degrade 20 μg K30 capsule in 1 h (**Figure 4C**). K30 gp42 did not detectably degrade capsule, and combining K30 gp42 with K30 gp41 in different molar ratios provided no increase in reactivity (data not shown) though the two proteins appeared to bind when mixed (Supplementary Figure S2). Quantitative assays confirmed the high activity of K5 depolymerase (260–850 nmol glucose equivalents released per min per mg protein), low activity of K30 gp41 (35–60 nmol per min per mg protein) (**Figure 4D**), and no activity of K30 gp42 (not shown).

### Depolymerase Sensitization of Bacteria to Serum Killing

**FIGURE 5:**
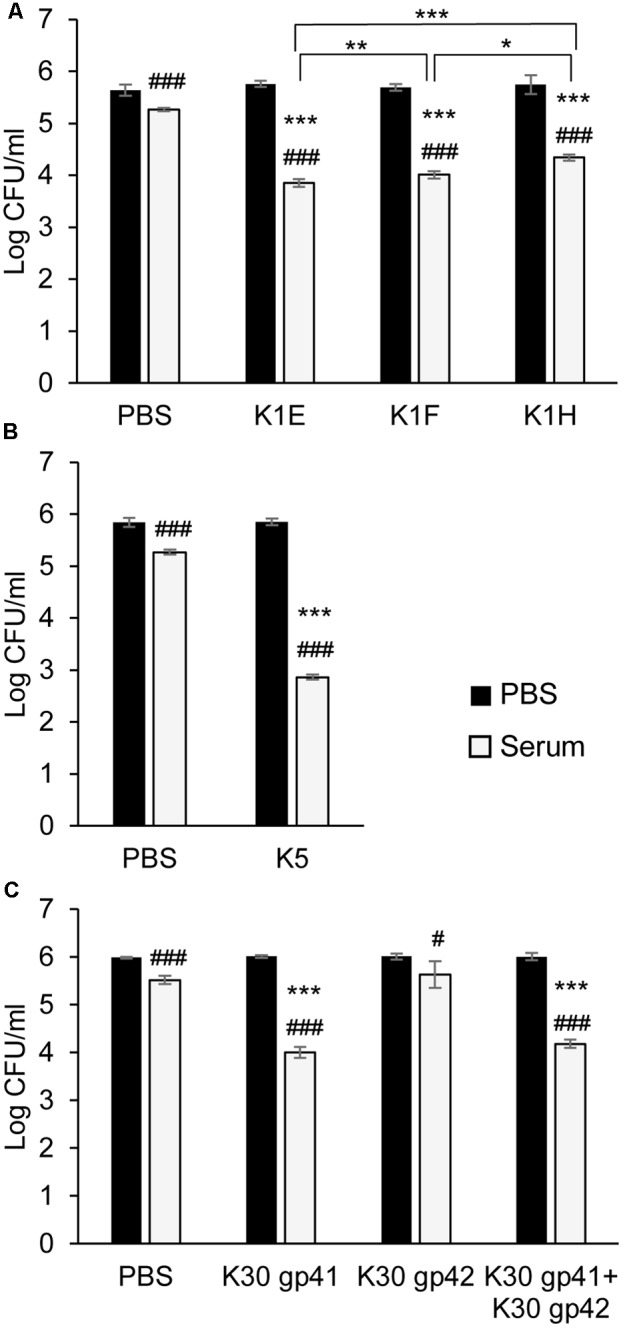
Serum sensitivity assay. 4 × 10^7^ CFU of *E. coli* RS218 **(A)**, ATCC 23506 **(B)**, or 6 × 10^7^ of *E. coli* E69 **(C)** were incubated with or without their respective depolymerases (100 μg/ml) for 1.5–2 h at 37°C. The mixtures were diluted and 4–6 × 10^4^ cells were treated with 75% serum for 1.5–2 h at 37°C before plating to determine CFU. Assays were repeated at least three times. The serum’s bactericidal effect was analyzed by Student’s *t*-test to compare cell survival in serum to that in PBS within each treatment: ^#^*p* < 0.05, ^###^*p* < 0.001. The enzyme’s effect on serum sensitivity of the bacteria was analyzed by Student’s *t*-test to compare the ratio of cell survival in serum over survival in PBS between different enzyme treatments or the control (PBS): ^∗^*p* < 0.05, ^∗∗^*p* < 0.01, ^∗∗∗^*p* < 0.001 compared to control or as noted.

Depolymerases can strip capsules and expose the underlying bacterium to immune attack such as complement-mediated killing (Roberts, 1996; Finlay and McFadden, 2006). We therefore tested our purified proteins using *in vitro* serum sensitivity assays. In the absence of serum, none of the depolymerases affected bacterial survival. Serum alone had a small effect in killing (**Figure 5**), while heat-inactivated serum slightly increased bacterial numbers (Supplementary Figure S3). For K1 bacteria, enzyme plus serum decreased bacterial survival by at least an order of magnitude (**Figure 5A**), with >10^4^ killing if the incubation time was extended to 3 h (not shown). As in the capsule degradation assays, K1E exhibited similar activity to K1F depolymerase, and both were superior to K1H in sensitizing bacteria to serum (**Figure 5A**).

K30 gp41 depolymerase, and especially K5 depolymerase, also sensitized bacteria to serum (**Figures 5B,C**). K30 gp42 alone had no significant effect on bacterial survival (with or without serum), and failed to provide any synergistic effect when combined with K30 gp41 (**Figure 5C**).

### Oligomerization of Purified Depolymerases

**FIGURE 6:**
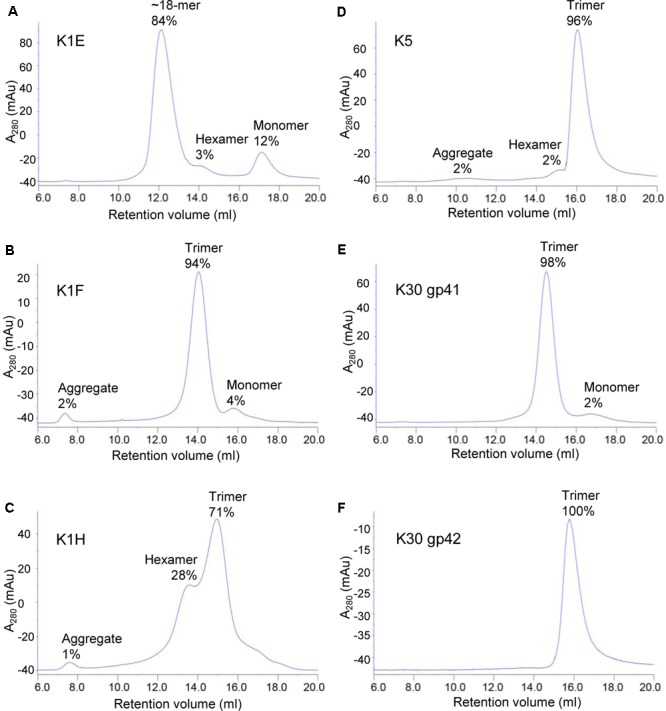
Size exclusion chromatography of purified depolymerases. 1 mg K1E **(A)**, 200 μg K1F **(B)**, 500 μg K1H **(C)**, 500 μg K5 **(D)**, 200 μg K30 gp41 **(E)** or 200 μg K30 gp42 **(F)** was loaded to a Superose 6 10/300 GL column for chromatographic analysis. mAu, micro absorbance unit. Molecular weight of each peak was estimated using calibration standards (GE Healthcare). The estimated multimeric status and percentage are indicated.

Most depolymerases are homotrimers, and one possible explanation for the discrepancy between the poor therapeutic performance of K1E in mice but good *in vitro* activity is that the purified proteins did not correctly trimerize. Analytical size exclusion chromatography of the depolymerases indicated that the K1E enzyme was mostly present as an ∼18-mer with only a trace of an apparent trimer (**Figure 6A**), while the K1F and K1H enzymes, and those from K5 and K30, were mostly trimeric (**Figures 6B–F**). Multimerization of K1E may limit its *in vivo* distribution following intramuscular injection, as further discussed below.

## Discussion

Capsule depolymerases are a promising class of new and non-traditional antibiotics. They have potential advantages over phage therapy: a broader host range than the phages that encode them, and an avoidance of bacterial lysis with concomitant endotoxin release. One downside is that they are active only on specific capsules. To our knowledge, seven phage-encoded depolymerases have now been shown to rescue laboratory rodents from bacterial infections (Mushtaq et al., 2004, 2005; Lin et al., 2014; Pan et al., 2015; and this work). These studies should motivate further investigations: (i) Does the approach generalize to any phage-derived capsular depolymerase? (ii) Can effective depolymerases be isolated from other environmental microorganisms? (iii) Can *in vitro* assays be developed that would serve as a predictor of *in vivo* activity?

This study addressed the general efficacy of phage-derived depolymerases as potential therapeutics by testing three distinct K1 depolymerases, plus similar enzymes from phages K5 and K30. We have shown that using depolymerases for treating bacterial infections is likely a generalizable and feasible therapeutic option, at least in the context of some infection models. Although we have not carefully optimized concentrations, 20 μg K1 or K5 depolymerase delivered intramuscularly into mice (∼1 mg/kg body weight) was sufficient to rescue mice from an otherwise lethal infection. This concentration is well within the range necessary for a practical human or other mammalian therapeutic. The K30 enzyme was first purified from K30 lysates as a complex of two proteins at 90 and 52 kD (McCallum et al., 1989). K30 gp41 and K30 gp42 appear to be the only two logical candidates at these sizes. However, K30 gp41 was sufficient and experienced no increased activity by the presence of K30 gp42, although the two proteins were purified separately. It is possible that co-expression, as occurs during phage infection, would yield improved activity. However, the N-terminal domain of K30 gp41 is homologous to that of the tail-binding domain of the T7 tail fiber, and it is possible that the interaction with K30 gp42 is more for binding the latter protein to the K30 virion rather than for improved enzymatic efficiency.

Besides testing the general therapeutic efficacy of capsule depolymerases, this study is also the first one to compare depolymerases of different origin against the same capsule type or bacterial strain *in vivo*. An unexpected result was that only two of the three K1 depolymerases, provided by intramuscular delivery, performed well in rescuing mice from a lethal bacterial infection. K1E did not, which was surprising for several reasons: (i) the enzyme worked well in previous work where a different, neonatal rat, model was used with gastrointestinal administration of bacteria (Mushtaq et al., 2004, 2005), (ii) K1E phage worked well *in vivo* using the same infection protocol (Bull et al., 2012), and (iii) K1F and K1H enzymes both worked well. To identify the basis of K1E depolymerase inferiority in the mouse infection model, *in vitro* activity assays were conducted, which showed that K1E depolymerase is at least equally efficient as the other K1 enzymes. Thus the *in vitro* assays failed to explain the *in vivo* inferiority of K1E.

Size exclusion chromatography may have revealed the cause of the discrepancy. The purified K1E depolymerase appeared as an 18mer, unlike other enzymes, which were mostly trimers. This observation is consistent with other reports that K1E depolymerase tends to aggregate (Hallenbeck et al., 1987; Gerardy-Schahn et al., 1995) although our preparation remains a soluble species. Phage tailspikes often require chaperones to fold correctly (Muhlenhoff et al., 2003; Schwarzer et al., 2007; Leiman and Molineux, 2008). The 38 kDa phage adaptor protein K1E gp37, which attaches the K1E depolymerase to the phage virion (Tomlinson and Taylor, 1985; Gerardy-Schahn et al., 1995; Stummeyer et al., 2006), may contribute to forming a specific trimeric species. It seems possible that multimers of a trimeric K1E depolymerase, although retaining activity *in vitro*, are unable to be efficiently disseminated *in vivo* following intramuscular injection. A preliminary test using intraperitoneal injection of 20 μg depolymerase following thigh injection of *E. coli* RS218 showed that K1E enzyme had good efficacy in treatment (data not shown). This observation together with the size exclusion chromatography suggests that poor dissemination of the K1E enzyme when administered intramuscularly may be the cause of the discrepancy. These variations highlight the difficulty of predicting therapeutic efficacy in humans from *in vitro* studies or from different rodent models, especially when using different routes of administration.

This study, like many others with a similar goal of treating an acute and lethal infection, administered the therapeutic agent at the same time as the pathogenic bacteria. Even though the depolymerases were injected into the opposite thigh of the mouse and thus had to diffuse or be transported to where the bacteria were growing, this “simultaneous” treatment is clearly not representative of natural therapeutic interventions. Treatment success with simultaneous administration is an important first step, but may not be a sufficient criterion for successful therapy in a natural setting. Our current investigations are testing the therapeutic efficacy of depolymerases after a designed delay in initiating treatment, which would better reflect actual clinical therapeutics. Tests of depolymerases in other infection environments, i.e., using additional model systems, are also now warranted.

Nonetheless, this study shows general efficacy of capsule depolymerases. These enzymes may thus provide an alternative to phage therapy *sensu stricto*, one with a broader host range than the source phages themselves. For instance, phage K1F does not grow on *E. coli* RS218; however, K1F depolymerase worked well in rescuing mice from a lethal dose of the bacterium. This study also shows that different depolymerases for the same capsule type may perform differently in certain settings. It may therefore be useful to have multiple sources of enzymes, which although structurally similar have different amino acid sequences, for the same capsule type. This would be particularly valuable in the event that patients develop immune responses to one. Environmental bacteria and other types of microbes can provide additional sources of depolymerases (Avery and Dubos, 1931; Dubos and Avery, 1931; Negus and Taylor, 2014). At a minimum, non-phage sources of depolymerases would further expand the possible sources of such enzymes, but they also offer the possibility of broader host range depolymerases and of evolving better activities (Bull and Gill, 2014).

## Ethics Statement

This study was carried out in accordance with the recommendations of NIH (National Institutes of Health) guidelines. The protocol (AUP-2015-00035) was approved by the University of Texas IACUC (Institutional Animal Care and Use Committee).

## Author Contributions

HL, JB, and IM designed the experiments. HL, MP, and JB carried out the animal experiments, and HL carried out all the other experiments. All authors analyzed data and wrote the manuscript.

## Conflict of Interest Statement

The authors declare that the research was conducted in the absence of any commercial or financial relationships that could be construed as a potential conflict of interest.
