# Supplementary material for: Therapeutic Application of Phage Capsule Depolymerases against K1, K5, and K30 Capsulated E. coli in Mice
Source: Front Microbiol. 2017 Nov 16;8:2257. doi: 10.3389/fmicb.2017.02257 (PMC5696595; doi:10.3389/fmicb.2017.02257)
Supplement: Supplementary file 1 [file Table_1.DOCX]

**Table S1. Primers for depolymerase cloning.**

| Primer | Sequence | Gene | Accession | GeneID | Protein_id |
| --- | --- | --- | --- | --- | --- |
| K1E-GibsonF | 5’AGCAGCGGCCTGGTGCCGCGCGGCAGCCATATGATTCAAAGACTAGGTTCTTCATTAGTT3’ | K1E | NC_007637 | 3837325 | YP_425027.1 |
| K1E-GibsonR | 5’TGCGGCCGCAAGCTTGTCGACGGAGCTCGATTAACTGATTTTATTAGTGGCATACATCTC3’ | K1E |  |  |  |
| K1F-GibsonF | 5’CTGGTGCCGCGCGGCAGCCATATGTCCACGATTACACAATTCC3’ | K1F | NC_007456 | 3707741 | YP_338127.1 |
| K1F-GibsonR | 5’CGCAAGCTTGTCGACGGAGCTCGATTACTTCTGTTCAAGAGCAGAA3’ | K1F |  |  |  |
| K1H-GibsonF | 5’CTGGTGCCGCGCGGCAGCCATATGTCAAGCGGATGCGGTGACGTTTTAA3’ | K1H | NC_027994 | 26040756 | YP_009168860.1 |
| K1H-GibsonR | 5’CGCAAGCTTGTCGACGGAGCTCGATTATTTATCTTCTAGTGCTGCCAGCC3’ | K1H |  |  |  |
| K5-GibsonF | 5’AGCAGCGGCCTGGTGCCGCGCGGCAGCCATATGGCTAAATTAACCAAACCTAATACTGAAGGAA3’ | K5 | NC_008152 | 5075948 | YP_654147.1 |
| K5-GibsonR | 5’TGCGGCCGCAAGCTTGTCGACGGAGCTCGATTACTTAGGCAGGGAAGCTAGTGCTTCC3’ | K5 |  |  |  |
| K30 gp41-GibsonF | 5’CTGGTGCCGCGCGGCAGCCATATGGACCAAGACATTAAAACAGTC3’ | K30 gp41 | NC_015719 | 10894528 | YP_004678762.1 |
| K30 gp41-GibsonR | 5’CGCAAGCTTGTCGACGGAGCTCGATTAGTGGACGCCGATTGTGTA3’ | K30 gp41 |  |  |  |
| K30 gp42-GibsonF | 5’CTGGTGCCGCGCGGCAGCCATATGTTAGACAAATTGAATCAGCC3’ | K30 gp42 | NC_015719 | 10894529 | YP_004678763.1 |
| K30 gp42-GibsonR | 5’CGCAAGCTTGTCGACGGAGCTCGATTATGCCCCAAAGGTGTCCAGTTTGA3’ | K30 gp42 |  |  |  |

**Table S2. Enzyme kinetics of K1 depolymerases.**

| Enzyme | K_M_ (μM) | k_cat_ (s^-1^) | k_cat_/K_M_ (μM^-1^ s^-1^) |
| --- | --- | --- | --- |
| K1E | 4.16 | 1.18 | 0.28 |
| K1F | 8.61 | 2.45 | 0.28 |
| K1H | 11.66 | 2.43 | 0.21 |

10 μg/ml of K1 enzymes were incubated with different K1 capsule concentrations for 30 min at 37 ℃. The resulting reducing sugar was quantified using DNSA. A Hanes-Woolf plot (a/v against a, where a is the substrate concentration and v is the reaction velocity) was made to estimate enzyme kinetic parameters: K_M_, Michaelis–Menten constant; k_cat_, turnover number; k_cat_/K_M_, catalytic efficiency.
