# Supplementary material for: Therapeutic Application of Phage Capsule Depolymerases against K1, K5, and K30 Capsulated E. coli in Mice
Source: Front Microbiol. 2017 Nov 16;8:2257. doi: 10.3389/fmicb.2017.02257 (PMC5696595; doi:10.3389/fmicb.2017.02257)
Supplement: Supplementary file 2 [file Image_1.PDF]

Supplementary Figures

Figure S1

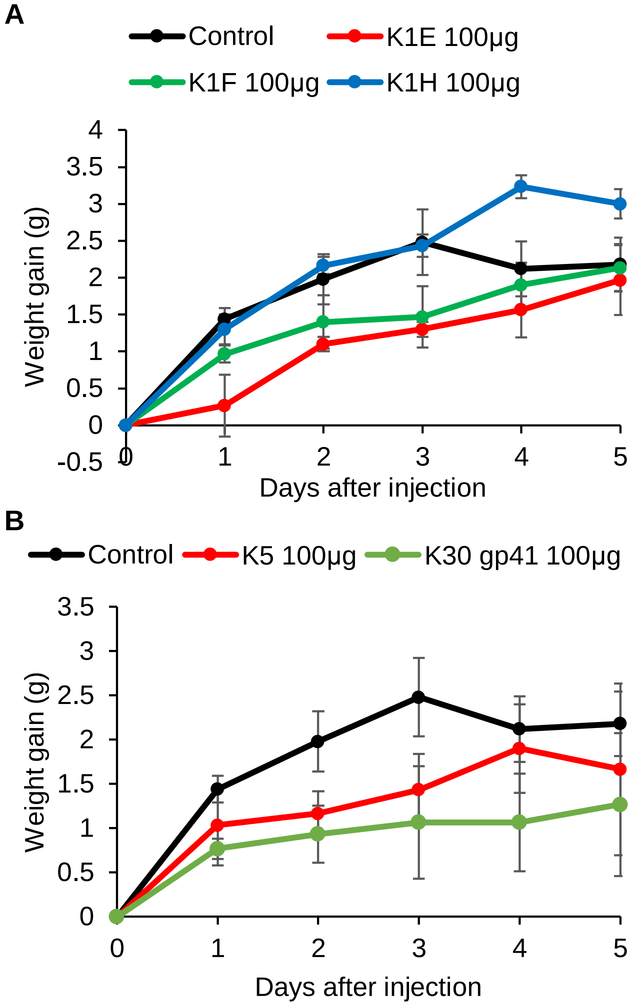

Figure S1. Body weight gains of mice receiving high dose depolymerase. 100 µg of each depolymerase or PBS as the control was injected to the right thighs of 3 - 5 mice. Survival and body weight were monitored for 5 days after injection. Body weight gain of each group as mean ± standard deviation was plotted over the time course. Statistics was performed using mixed ANOVA with repeated measures by SPSS software.

Figure S2

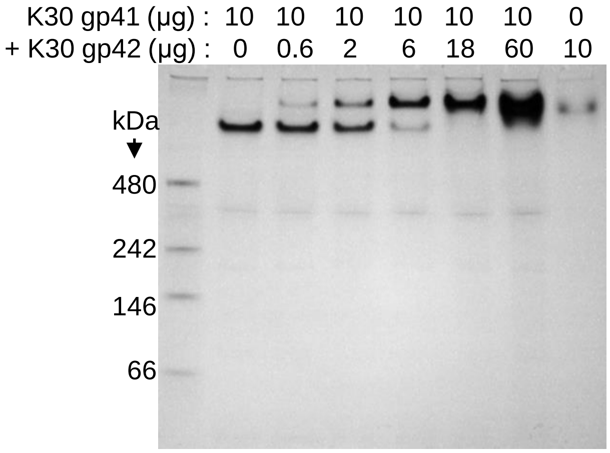

Figure S2. Native PAGE of K30 gp41 and K30 gp42 mixtures. K30 gp41, K30 gp42 or their mixture at different molar ratios (10 µg : 0.6 µg ~ molar 10 : 1, 10 µg : 2 µg ~ molar 3 : 1, 10 µg : 6 µg ~ molar 1 : 1, 10 µg : 18 µg ~ molar 1 : 3, 10 µg : 60 µg ~ molar 1 : 10) was loaded to native PAGE without heating, along with NativeMark protein standard (Life Technologies). Native PAGE followed essentially the same procedure as SDS-PAGE except the exclusion of SDS from the buffers. After electrophoresis, proteins were stained with Coomassie brilliant blue.

Figure S3

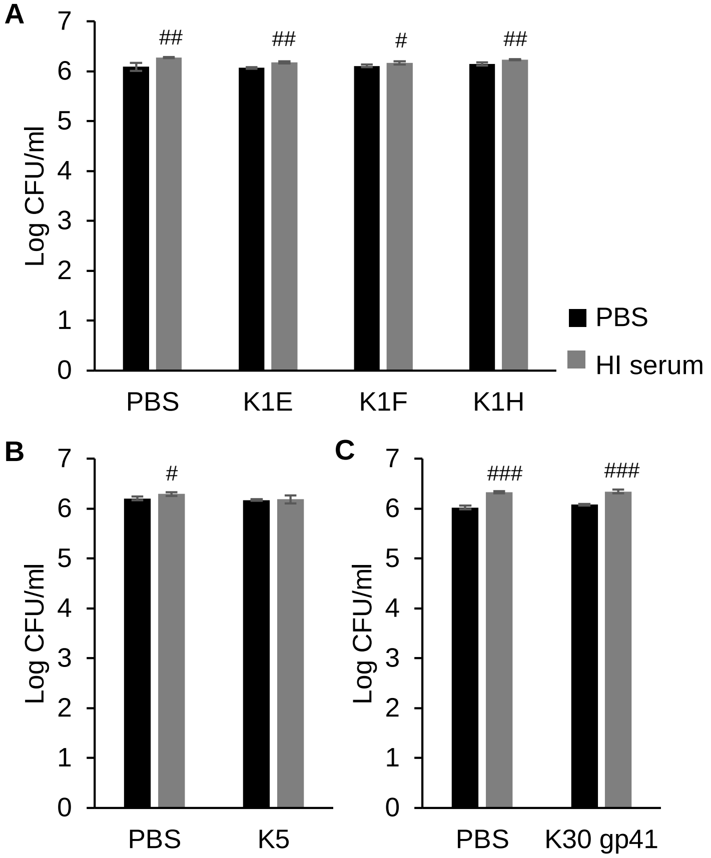

Figure S3. Serum sensitivity assay with heat inactivated serum. The same procedure as Figure 5 was followed but using heat inactivated (HI) serum instead of serum. Assays were repeated at least three times. The HI serum's effect on cell survival was analyzed by Student's t-test to compare cell survival in HI serum to that in PBS within each treatment: #$p<0.05$, ##$p<0.01$, ###$p<0.001$. The enzyme's effect on bacterial survival in HI serum was analyzed by Student's t-test to compare the ratio of cell survival in serum over survival in PBS between different enzyme treatments or the control (PBS): no statistical significance.
